# Editorial: Measuring resting cerebral perfusion using magnetic resonance imaging (MRI)

**DOI:** 10.3389/fphys.2024.1447417

**Published:** 2024-07-08

**Authors:** James Duffin, Alex A. Bhogal

**Affiliations:** ^1^ Department of Anaesthesia and Pain Management, University of Toronto, Toronto, ON, Canada; ^2^ Department of Physiology, University of Toronto, Toronto, ON, Canada; ^3^ Thornhill Research Inc., Toronto, ON, Canada; ^4^ Center for Image Sciences, Department of Radiology, University Medical Center Utrecht, Utrecht, Netherlands

**Keywords:** perfusion, oxygenation, MRI, DSC, arterial spin label (ASL) MRI, calibrated BOLD

## Cerebral perfusion

Cerebral perfusion metrics such as cerebral blood flow (CBF) provide an important assessment of cerebrovascular health and the hemodynamic consequences of cerebrovascular disease and ischemic events. For example, the detection of collateral blood circulation, which is widely recognised as a crucial protective mechanism that can significantly influence clinical outcomes following ischemic events such as stroke. Moreover, understanding cerebral blood flow distribution patterns can aid in surgical planning for interventional procedures, and assessment of their effectiveness. These considerations underscore the necessity for developing MRI-based techniques to measure perfusion metrics together with their associated post-processing methods. An advantage of MRI is the ability to deliver volumetric, temporally resolved information to which classical methods such as transcranial doppler are insensitive as Fico et al. explain. They demonstrated this aspect, showing that 4D flow MRI was more sensitive to age-related differences in cerebrovascular reactivity when comparing with analogous velocity-based measurements made using TCD. This was despite good agreement between middle cerebral artery velocity measurements as measured using each technique.

The reproducibility of MRI measurements is an important consideration when examining longitudinal changes in perfusion metrics, as Madsen et al. remind us. They examined the longitudinal reproducibility of a selection of metabolic (lactate and N-acetyl-aspartate (NAA) concentrations) and hemodynamic (cerebral blood flow, cerebral metabolic rate of oxygen consumption and global arterio-venous oxygen saturation difference) MR parameters. With the exception of lactate, MR parameters showed good within-day reproducibility, which declined between 7 days and several week measurements. Understanding physiological variability is crucial for optimizing study designs and establishing the sample sizes required to adequately power large-scale studies.

## Novel methods of measuring cerebral perfusion

Dynamic susceptibility contrast (DSC) in MRI is a widely used method for perfusion imaging. Current clinical DSC-MRI involves the injection and tracking of a gadolinium (Gd) based contrast agent. However, there are safety concerns associated with Gd (Runge, 2000), as it has been shown to accumulate in the body (Kanda et al., 2014; Gulani et al., 2017) and our drinking water (Rogowska et al., 2018). Gd use is also limited in the sense that measurements are not repeatable within the same scan session since the injection can only be performed once. A novel alternative to Gd contrast is to establish an endogenous contrast bolus consisting of deoxyhemoglobin (dOHb), which is also paramagnetic. This can be done by means of a transient desaturation of arterial hemoglobin using a transient hypoxic respiratory challenge. Such an approach leads to a similar susceptibility change as Gd but is produced non-invasively. The dOHb bolus can be tracked using the same gradient-echo sequences, and as Stumpo et al. demonstrated the resulting images are in good agreement with those obtained using conventional DSC methods.

The nature of the dOHb stimulus has also provided the opportunity to apply existing analysis methods developed for hypercapnic respiratory challenges to calculate voxel-wise perfusion metrics. One such method is transfer function analysis, and Sayin et al. showed that it can also be applied to transient hypoxia-induced changes in dOHb to provide perfusion information. The use of dOHb as a novel non-invasive contrast agent is clearly promising.

Interestingly, the search for non-invasive techniques has encouraged several studies that derive perfusion-like metrics using CO_2_ as a stimulus. Vu et al. implemented sinusoidal CO_2_ challenges for concurrent assessment of relative cerebral blood flow and cerebral vascular reactivity (CVR) while Fitzgerald et al. exploit the sharp signal changes associated with CO_2_-induced vasodilation to derive blood transit times in grey matter tissue.

However, there is an important caveat pertaining to the use of a vascular stimulus such as CO_2_ for obtaining perfusion metrics. Increasing CO_2_ with its associated CBF increase alters the ‘baseline perfusion’ state. To counteract this aspect, multi-delay arterial spin labelling methods are appealing. Pinto et al. showed that by accounting for macrovascular signal components and dispersion effects, more accurate quantification of CBF could be achieved. Similarly, Shah et al. used imputation modelling to correct measurement errors for improved CBF assessment using phase contrast MR in large cerebral vessels.

In addition to advanced modelling methods, clever acquisition strategies to maximize spatial resolution as were employed by Kashyap et al. These approaches will help advance ASL towards greater clinical adoption; particularly multi PLD variants. Indeed, Krishnamurthy et al. already showcase applications for understanding stroke etiology using ASL, while Jellema et al. highlight applications for perfusion (and complimentary) intra-operative acquisition strategies in the even the most sensitive paediatric brain tumour patients.

### Measuring blood oxygenation by MRI

Finally, we must emphasize that perfusion is only one aspect of cerebral regulation, and it is closely linked brain metabolic processes which are essential for maintaining homeostasis. As suggested by Le et al., characterizing the interplay between blood oxygen delivery and tissue consumption can pave the way for novel biomarkers of microvascular disease. The work of Williams et al. also helps to better interpret functional MRI studies that often rely on the Blood Oxygenation Level Dependent (BOLD) signal contrast that originates from changes in perfusion mediated by the cerebrovascular reactivity response coupled with changes in oxygen consumption. Understanding these relationships will be key to using advanced physiological MRI towards routine clinical practice.

## Conclusion

In summary, the collection of papers in this Research Topic highlight the importance of cerebral perfusion metrics in assessing cerebrovascular health. They demonstrate that there are many ways of making such measurements. Some require invasive techniques, such as the intravenous injection of the paramagnetic contrast agent Gadolinium, and others are non-invasive, such as utilizing changes in dOHb produced by controlling respired gases. They also show that improving measurement techniques and data analysis methodologies offer ways of improving the determination of resting perfusion metrics. We, the editors of this CVR Research Topic, hope that readers will benefit from the collection of articles presented. We believe that MRI perfusion metrics will become ever more useful in the assessment of cerebrovascular disease in clinical practice.

## References

[B1] Gulani V. Calamante F. Shellock F. G. Kanal E. Reeder S. B. International Society for Magnetic Resonance in M (2017). Gadolinium deposition in the brain: summary of evidence and recommendations. Lancet Neurol. 16, 564–570. 10.1016/S1474-4422(17)30158-8 28653648

[B2] Kanda T. Ishii K. Kawaguchi H. Kitajima K. Takenaka D. (2014). High signal intensity in the dentate nucleus and globus pallidus on unenhanced T1-weighted MR images: relationship with increasing cumulative dose of a gadolinium-based contrast material. Radiology 270, 834–841. 10.1148/radiol.13131669 24475844

[B3] Rogowska J. Olkowska E. Ratajczyk W. Wolska L. (2018). Gadolinium as a new emerging contaminant of aquatic environments. Environ. Toxicol. Chem. 37, 1523–1534. 10.1002/etc.4116 29473658

[B4] Runge V. M. (2000). Safety of approved MR contrast media for intravenous injection. J. magnetic Reson. imaging 12, 205–213. 10.1002/1522-2586(200008)12:2<205::aid-jmri1>3.0.co;2-p 10931582

